# Sequence-based breeding; application to unravel traits of the 6F crops: food, feed, fiber, flowers, fuel and fun

**DOI:** 10.1186/1753-6561-5-S7-O19

**Published:** 2011-09-13

**Authors:** Mark van Haaren

**Affiliations:** 1 Keygene Inc, the Netherlands

## 

The genomes of the world’s most important crop species differ enormously in size, ploidy levels, repeat composition and germplasm diversity, posing a tremendous challenge to utilize them for breeding and trait improvement. Keygene’s Crop Genome Center addresses this challenge by developing and applying sequence-based methodologies to advance building genome assemblies and elucidating genetic diversity of 6F crops. For example, novel strategies to build very high quality genome sequence assemblies make use of our Whole Genome Profiling (WGP) technology and high parallel local sequence assemblies. For sequence-based genotyping we developed random Sequence-Based Genotyping (rSBG), a technology which incorporates the high-throughput sequencing capacity of the Illumina Genome Analyzer (GA)-*II* and the genome complexity reduction capabilities of AFLP^®^. rSBG allows for simultaneous sequence-based marker discovery and detection and is therefore a highly suitable technology for cost efficient, highly flexible, sequence-based genotyping, not requiring prior sequence information and custom reagents. These technologies, together with KeyGene's lead discovery and molecular mutagenesis platforms, significantly increase the rate with which the genetic basis for (complex) traits can be unraveled. In this presentation, I will present recent applications of these technologies in our breeding research programs.

The AFLP^®^, Whole Genome Profiling and rSBG technologies are covered by patents and patent applications owned by Keygene N.V.

AFLP is a registered trademark of Keygene N.V.

